# Antiangiogenic and anticolorectal cancer effects of metronomic irinotecan chemotherapy alone and in combination with semaxinib

**DOI:** 10.1038/sj.bjc.6604352

**Published:** 2008-04-29

**Authors:** G Bocci, A Falcone, A Fioravanti, P Orlandi, A Di Paolo, G Fanelli, P Viacava, A G Naccarato, R S Kerbel, R Danesi, M Del Tacca, G Allegrini

**Affiliations:** 1 Division of Pharmacology and Chemotherapy, Department of Internal Medicine, University of Pisa, Pisa, Italy; 2 Division of Medical Oncology, General Hospital of Livorno, Department of Oncology, University of Pisa, Pisa, Italy; 3 Division of Surgical, Molecular and Ultrastructural Pathology, Department of Oncology, University of Pisa, Pisa, Italy; 4 Molecular and Cellular Biology Research, Sunnybrook Health Sciences Centre, Toronto, Ontario, Canada; 5 Department of Medical Biophysics, University of Toronto, Toronto, Ontario, Canada

**Keywords:** metronomic chemotherapy, angiogenesis, irinotecan, semaxinib, colon cancer

## Abstract

Metronomic chemotherapy refers to the administration of chemotherapy at low, nontoxic doses on a frequent schedule with no prolonged breaks. The aim of the study is to rationally develop a CPT-11 metronomic regimen in preclinical settings of colon cancer. *In vitro* cell proliferation, apoptosis and thrombospondin-1/vascular endothelial growth factor (TSP-1/VEGF) expression analyses were performed on endothelial (HUVEC, HMVEC-d) and colorectal cancer (HT-29, SW620) cells exposed for 144 h to metronomic concentrations of SN-38, the active metabolite of CPT-11. HT-29 human colorectal cancer xenograft model was used, and tumour growth, microvessel density and VEGF/TSP-1 quantification was performed in tumours. *In vitro* and *in vivo* combination studies with the tyrosine inhibitor semaxinib were also performed. SN-38 preferentially inhibited endothelial cell proliferation alone and interacted synergistically with semaxinib; it induced apoptosis and increased the expression and secretion of TSP-1. Metronomic CPT-11 alone and combined with semaxinib significantly inhibits tumour growth in the absence of toxicity, which was accompanied by decreases in microvessel density and increases in TSP-1 gene expression in tumour tissues. *In vitro* results show the antiangiogenic properties of low-concentration SN-38, suggesting a key role of TSP-1 in this effect. *In vivo*, the CPT-11 metronomic schedule is effective against tumour and microvessel growth without toxic effect on mice.

The interest in exploiting chemotherapeutic drugs for their antiangiogenic properties in the context of cancer therapy has been stimulated by a number of preclinical studies, in particular the pioneering contribution of Folkman and co-workers and that of the groups of Kerbel and Hanahan (Browder *et al*, 2000; Klement *et al*, 2000; Bocci *et al*, 2002; Man *et al*, 2002; Pietras and Hanahan, 2005) using drugs such as cyclophosphamide, oral fluoropyrimidines, vinblastine or taxanes at doses much lower than the maximum-tolerated dose (MTD), administered at close regular intervals either alone or in combination with targeted antiangiogenic drugs. The metronomic chemotherapy strategy – a long-term, low-dose, frequent administration of chemotherapeutic drugs with no prolonged drug-free breaks (Kerbel and Kamen, 2004) – appears to enhance the efficacy of the antiangiogenic effects of standard chemotherapy (with lower toxic effects) and implies that activated vascular endothelial cells (Bocci *et al*, 2002) or circulating endothelial progenitors (Bertolini *et al*, 2003) may be more sensitive to low or lower doses of various chemotherapeutic drugs. Besides a direct effect on endothelial cell proliferation, protracted exposure to low concentrations of several different anticancer agents, including microtubule inhibitors and alkylating agents, can cause marked induction, *in vitro* and *in vivo,* of gene and protein expression of thrombospondin-1 (TSP-1), a potent and endothelial-specific inhibitor of angiogenesis (Sund *et al*, 2005), suggesting that it could be a key mediator for metronomic chemotherapy activity (Bocci *et al*, 2003; Damber *et al*, 2006; Hamano *et al*, 2004).

Despite abundant information about the pharmacology of irinotecan (CPT-11) (Di Paolo *et al*, 2006) and its active metabolite SN-38 on cancer cells using different therapeutic approaches, no data are currently available about the preclinical effects of metronomic CPT-11 administration on pathological angiogenesis and tumour growth. O'Leary *et al* (1999) have described an antiangiogenic effect of CPT-11 in a single experimental setting (using a cornea model of angiogenesis), whereas Kamiyama *et al* (2005) reported that SN-38, at cytotoxic standard doses, inhibited both endothelial cell proliferation and tube formation, decreasing HIF-1*α* and vascular endothelial growth factor (VEGF) gene expression in glioma cells under both normoxic and hypoxic conditions. However, no further preclinical studies have followed these preliminary reports evaluating possible additional mechanisms of drug action.

On the basis of this background, we decided to test, for the first time, the hypothesis that CPT-11 metronomic regimens can be effective in preclinical settings of colon cancer treatment; specifically, we investigated the *in vitro* and *in vivo* antiangiogenic/antitumour activity and the modulation of pro- (VEGF) and antiangiogenic (TSP-1) factor expression/secretion using low-dose schedules. Moreover, the effect of the simultaneous combination of irinotecan or SN-38 with semaxinib was studied both *in vitro* and *in vivo*.

## MATERIALS AND METHODS

### *In vitro* studies

#### Materials, drugs and cell lines

Recombinant human epidermal growth factor (EGF), basic fibroblast growth factor (bFGF) and VEGF were from PeproTechEC LTD (London, UK). Cell culture media, MCDB131 and DMEM, were purchased from Gibco BRL (Paisley, UK), quantitative real-time PCR (QRT-PCR) reagents were from Applied Biosystems (Foster City, CA, USA), supplements and all other chemicals not listed in this section were obtained from Sigma Chemical Co. (St Louis, MO, USA).

SN-38 was a generous gift from Pfizer (Groton, CT, USA) and dissolved in a stock solution of 10 mM in 100% DMSO for *in vitro* studies. CPT-11 (Pfizer) was purchased from the University hospital pharmacy (Ospedale S Chiara, Pisa, Italy) and reconstituted as per the manufacturer's instructions to a stock concentration of 20 mg ml^−1^ by the addition of sterile saline for *in vivo* studies.

Semaxinib (SU5416) was purchased from Sigma Chemical Co. and was dissolved in 100% DMSO for *in vitro* use and in a solution of 99% PEG-300 (w/v) and 1% Tween 80 for *in vivo* studies (Bocci *et al*, 2004a).

Human umbilical vein endothelial cells (HUVEC) and the human dermal microvascular endothelial cells (HMVEC-d; Clonetics, San Diego, CA, USA) were maintained in MCDB131 culture medium supplemented with 10% heat-inactivated FBS, L-glutamine 2 mM, heparin 10 U ml^−1^, EGF 10 ng ml^−1^ and bFGF 5 ng ml^−1^. The human colon tumour cell lines HT-29 and SW620 (ATCC, Manassas, MA, USA) were maintained in 10% FBS DMEM medium supplemented with L-glutamine 2 mM.

#### Cell proliferation assay and apoptosis measurements

Human umbilical vein endothelial cells, HMVEC-d, HT29 and SW620 cells were plated in 24-well sterile plastic plates (1% gelatin-coated for the endothelial cells) and treated continuously for 144 h (1 × 10^3^ and 0.5 × 10^3^ cells per well of normal or cancer cells, respectively, in 1 ml of medium) with various concentrations of SN-38 (1–100 000 pM) adding fresh solutions with new medium every 24 h (Bocci *et al*, 2002). Furthermore, to determine a time-dependent effect on endothelial cell proliferation, HMVEC-d cells were exposed for 72 h to SN-38 (1–100 000 pM). At the end of the experiments, cells were harvested with trypsin/EDTA and viable cells counted with a haemocytometre. Cell viability was assessed by trypan blue dye exclusion (Bocci *et al*, 2005). The data are presented as the percentage of the vehicle-treated cells. The concentration of drug that reduced cell proliferation by 50% (IC_50_) *vs* controls was calculated by nonlinear regression fit of the mean values of the data obtained in triplicate experiments (at least nine wells for each concentration).

To quantify the degree of apoptosis induced by the drug treatments, HMVEC-d, HUVEC, HT29 and SW620 cells were continuously treated for 144 h with SN-38 at a concentration corresponding to the experimental IC_50_ of cell proliferation (15, 200, 1500 and 650 pM, respectively) or vehicle alone. At the end of the incubation, cells were washed twice with PBS and fixed in 4% buffered paraformaldehyde for 15 min. Cells were resuspended and incubated for an additional 15 min in a solution containing 8 *μ*g ml^−1^ bisbenzimide HCl (Hsueh *et al*, 2000). Cells were spotted on glass slides and were examined by fluorescence microscopy (Leica, Wetzlar, Germany). A total of 100 cells from nine randomly chosen microscopic fields were counted, and the percentage of cells displaying chromatin condensation and nuclear fragmentation relative to the total number of counted cells (apoptotic index) was calculated.

#### *In vitro* assessment of synergism between semaxinib and SN-38 on endothelial cells

Semaxinib combined with SN-38 was explored on HMVEC-d cells with the simultaneous continuous exposure of semaxinib (0.01–100 *μ*M) plus SN-38 (1–10 000 pM) for 144 h at a fixed molar concentration ratio of 10 000 : 1. To evaluate the level of interaction (synergistic, additive or antagonist) between SN-38 and semaxinib, the method proposed by Chou (2006) was followed. Briefly, synergism or antagonism for semaxinib plus SN-38 was calculated on the basis of the multiple drug–effect equation, and quantitated by the combination index (CI), where CI<1, CI=1 and CI>1 indicate synergism, additive effect and antagonism, respectively. On the basis of the classic isobologram for mutually exclusive effects, the CI value was calculated as: 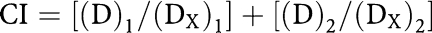 At the 50% inhibition level, (*D*_x_)_1_ and (*D*_x_)_2_ are the concentrations of semaxinib and SN-38, respectively that induce a 50% inhibition of cell growth; (*D*)_1_ and (*D*)_2_ are the concentrations of semaxinib and SN-38 in combination, which also inhibits cell growth by 50% (isoeffective as compared with the single drugs alone).

#### Real-time PCR analysis of human VEGF and TSP-1 gene expression on tumour and endothelial cells

To evaluate the expression of the human *VEGF* and *TSP-1* genes, 2 × 10^4^ HMVEC-d, HUVEC, HT29 and SW620 cells were grown in their respective media and treated with SN-38 at a concentration corresponding to the experimental IC_50_ of cell proliferation (15, 200, 1500 and 650 pM, respectively) and at a lower and inactive dose (1, 10, 10 and 10 pM, respectively) or with vehicle alone for 144 h. Briefly, RNA (1 *μ*g) was reverse transcribed as previously described (Bocci *et al*, 2005), the resulting cDNA was diluted (2 : 3) and then amplified by QRT-PCR with the Applied Biosystems 7900HT sequence detection system. Vascular endothelial growth factor and TSP-1 validated primers were purchased from Applied Biosystems (Assay ID Hs00170236_m1 and Hs00173626_m1, respectively). The PCR thermal cycling conditions and optimisation of primer concentrations were followed as per the manufacturer's instructions. Amplifications were normalised to glyceraldehyde 3-phosphate dehydrogenase (GAPDH), and the quantitation of gene expression was performed using the ΔΔ*C*_t_ calculation, where *C*_t_ is the threshold cycle; the amount of target, normalised to the endogenous control and relative to the calibrator (vehicle-treated control cells), is given as 2^−ΔΔ*Ct*^.

#### Human VEGF and TSP-1 detection in conditioned media by ELISA and EIA

Human dermal microvascular endothelial cells, HUVEC, HT29 and SW620 cells were grown in their respective media and treated with SN-38 at a concentration corresponding to the experimental IC_50_ of cell proliferation (15, 200, 1500 and 650 pM, respectively) and at a lower and inactive concentration (1, 10, 10 and 10 pM, respectively) or with vehicle alone for 144 h. Furthermore, to determine a time-dependent effect on endothelial cell secretion, HMVEC-d cells were exposed for 72 h to the SN-38 experimental IC_50_ of cell proliferation (13 nM). To measure secreted VEGF and TSP-1, at the end of the experiment the medium of each well was discarded and replaced with serum-free medium for 4 h. Each sample was then assayed for human VEGF and TSP-1 concentrations by the ELISA Kit Quantikine (R&D Systems, Minneapolis, MN, USA) and by the ChemiKine Human TSP-1 EIA Kit (Chemicon, Temecula, CA, USA), respectively, and normalised by total protein concentration. The optical density was determined using the microplate reader Multiskan Spectrum (Thermo Labsystems, Milan, Italy) set to 450 nm (with a wavelength correction set to 540 nm) for the VEGF kit and to 490 nm for TSP-1 kit. All experiments were repeated, independently, six times with at least nine samples for each concentration.

#### Akt (pThr^308^) ELISA assay

To detect the phosphorylation of Akt in endothelial cells after a short period of time (72 h), HMVEC-d cells were treated with SN-38 at a concentration corresponding to the experimental IC_50_ of cell proliferation (13 nM) or with vehicle alone for 72 h. To measure pAkt, at the end of the experiment, the cells were harvested and immediately frozen with liquid nitrogen. Each sample was then assayed for human pAkt (pThr^308^) concentrations by the ELISA Kit PhosphoDetect Akt (pThr^308^) (Calbiochem, San Diego, CA, USA) and normalised by total protein concentration. The optical density was determined using the microplate reader Multiskan Spectrum set to 450 nm. The results were expressed as pAKT unit per mg of total protein. All experiments were repeated, independently, six times with at least nine samples for each concentration.

### *In vivo* studies

#### Animals

CD nu/nu male mice, weighing 20–25 g, were supplied by Charles River (Milan, Italy) and were allowed unrestricted access to food and tap water. Housing and all procedures involving animals were performed according to the protocol approved by the Academic Committee for the animal experimentation of the University of Pisa, in accordance with the European Community Council Directive 86–609, recognised by the Italian government, on animal welfare. Each experiment employed the minimum number of mice needed to obtain statistically meaningful results.

#### HT-29 xenografts in nu/nu mice and drug treatments

HT-29 cell viability was assessed by trypan blue dye exclusion, and on day 0, 1.3 × 10^6^±5% cells per mouse were inoculated subcutaneously (s.c.) between the scapulae in 0.2 ml per mouse of culture medium without FBS. Animal weights were monitored and upon appearance of a subcutaneous mass, tumour dimensions were measured every 2 days in two perpendicular directions using callipers. Tumour volume (mm^3^) was defined as [(*w*_1_ × *w*_2_ × *w*_2_) × (*π*/6)], where *w*_1_ and *w*_2_ were the largest and the smallest tumour diameters (mm), respectively (Bocci *et al*, 2004b). The mice were randomised into groups of six animals. To treat an established tumour (∼35 mm^3^), from day 15 after cell inoculation, CPT-11 was administered i.p. as follows: (1) CPT-11 at the MTD of 100 mg kg^−1^ five times at 7-day intervals (Allegrini *et al*, 2004); (2) metronomic CPT-11 every day at the dose of 4 mg kg^−1^ for 50 days (a 72% decrease *vs* MTD dose); (3) a sequential treatment of an initial (‘up-front’) single dose of CPT-11 100 mg kg^−1^ followed by metronomic CPT-11 4 mg kg^−1^ every day for 49 days. The control group was injected i.p. with vehicle alone (saline solution). The experimental period ended 65 days after the inoculation of tumour cells whereas control mice were killed by an anaesthetic overdose when the tumour volume reached a mean value of 3200 mm^3^.

To establish the antitumour activity of metronomic CPT-11 and semaxinib combination *in vivo*, an additional experiment was performed. On day 15, from HT-29 cell inoculum, metronomic CPT-11 schedules and their simultaneous combinations with semaxinib were administered i.p. as follows: (1) metronomic CPT-11 every day at the dose of 4 mg kg^−1^ for 33 days; (2) an initial single dose of CPT-11 100 mg kg^−1^ followed by metronomic CPT-11 4 mg kg^−1^ every day for 32 days; (3) combination treatment of CPT-11 4 mg kg^−1^ every day and semaxinib 10 mg kg^−1^ twice weekly; (4) combination treatment of an initial single dose of CPT-11 100 mg kg^−1^ followed by metronomic CPT-11 4 mg kg^−1^ every day and semaxinib 10 mg kg^−1^ twice weekly. The control group was injected i.p. with vehicle alone (saline solution and PEG-300/Tween 80). Animal weights were monitored as described above. Drug efficacy was based on percentage of the average treated-tumour volume divided by the average vehicle-control-tumour volume (% *T*/*C*) (Tahir *et al*, 2003).

#### Immunohistochemistry, microvessel density and real-time RT-PCR on HT-29 tumour tissue samples

After surgical resection, tumour tissue samples from all the different treatment groups were split into two aliquots, one fixed in 10% phosphate-buffered formaldehyde for 12–24 h and embedded in paraffin for histology and immunohistochemistry, the other immediately frozen in liquid nitrogen for the analysis of human *VEGF* and *TSP-1* gene expression by real-time RT-PCR.

Five-micrometre sections were stained with haematoxylin–eosin for histological analysis. Adjacent sections were cut for immunohistochemistry as previously described (Viacava *et al*, 2004) using the following primary antibodies: rat anti-mouse CD31 (dilution, 1 : 100; PharMingen, San Diego, CA, USA) for evaluation of microvascular density and the rabbit anti-human VEGF-A to screen VEGF as a tumour cell-associated angiogenic factor (Oncogene Research Products, Cambridge, MA, USA; dilution 1 : 100). Negative controls were obtained by replacing the primary antibody with nonimmune serum.

Cytoplasmic staining was scored positive for VEGF-A. The degree of positivity was evaluated by calculating the percentage of immunoreactive cells on a minimum of 500 cells (Viacava *et al*, 2004). To calculate microvessel density, three most vascularised areas of the tumour (‘hot spots’) were selected and mean values obtained by counting vessels. A single microvessel was defined as a discrete cluster of cells positive for CD31 staining, with no requirement for the presence of a lumen. Microvessel counts were performed at × 200 ( × 20 objective lens and × 10 ocular lens; 0.74 mm^2^ per field). All parameters were determined independently by three expert pathologists (PV, AGN and GF), and discordant cases were solved by simultaneous review.

The frozen HT-29 xenografts were collected and total RNA was isolated from the tissues using the Trizol Kit (Gibco). Human *VEGF* and *TSP-1* gene expression in tumour samples was performed by real-time PCR as described above.

#### Statistical analysis

The analysis by ANOVA, followed by the Student–Newman–Keuls test, was used to assess the statistical differences of data *in vitro* and *in vivo*. *P*-values lower than 0.05 were considered significant. Statistical analyses were performed using the GraphPad Prism software package version 4.0 (GraphPad Software Inc., San Diego, CA, USA).

## RESULTS

### *In vitro* studies

#### Protracted low-dose treatment with SN-38 preferentially inhibits endothelial cell proliferation and induces apoptosis

**Figure 1:**
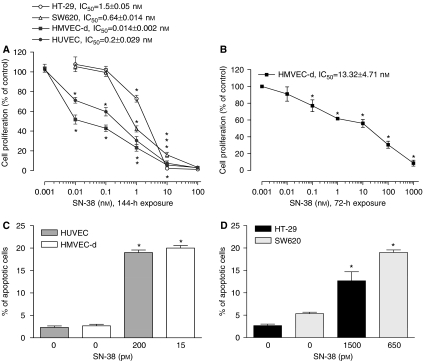
Effect of low-dose SN-38, the active metabolite of CPT-11, on *in vitro* cell proliferation (**A** and **B**). The antiproliferative effects of the drug were studied using prolonged continuous exposures (144 h) on HUVEC, HMVEC-d, HT-29 and SW620 cell lines (**A**) and shorter ones (72 h) on HMVEC-d cells (**B**). Symbols and bars, mean values±s.e., respectively. ^*^P<0.05 *vs* vehicle-treated controls. IC_50_, the concentration of drug that reduced cell proliferation by 50%. Pro-apoptotic effects of (**C**) SN-38 on proliferating endothelial cells treated at their experimental IC_50_s (lower concentrations) for 144 h and (**D**) SN-38 on proliferating colorectal cancer cells treated at their experimental IC_50_s (higher concentrations) for 144 h. Columns and bars, mean values ±s.e., respectively. ^*^P<0.05 *vs* vehicle-treated controls.

The 144-h SN-38 exposure inhibited the cell growth of HMVEC-d and HUVEC in a concentration-dependent manner (Figure 1A), and the calculated IC_50_ values were 0.014±0.002 and 0.2±0.029 nM, respectively; in contrast, SN-38 did not significantly affect the proliferation of SW620 and HT-29 cell line at low concentrations, showing much higher IC_50_ values (0.64±0.014 and 1.5±0.05 nM, respectively; Figure 1A). Furthermore, the 72-h treatment showed a lower antiproliferative activity of SN-38 on microvascular endothelial cells (IC_50_ 13.3±4.71 nM), underlying a time-dependent effect (Figure 1B).

As shown in Figure 1C, after 144 h of treatment at their respective SN-38 IC_50_s, a significant percentage of apoptotic endothelial cells in the treated samples were found (e.g. 20±0.5 *vs* 2.5±0.3% of control HMVEC-d apoptotic cells) when compared to controls. These apoptotic percentages were similar to those obtained and expected, when treating cancer cells at higher doses such as their IC_50_s (Figure 1D).

**Figure 2:**
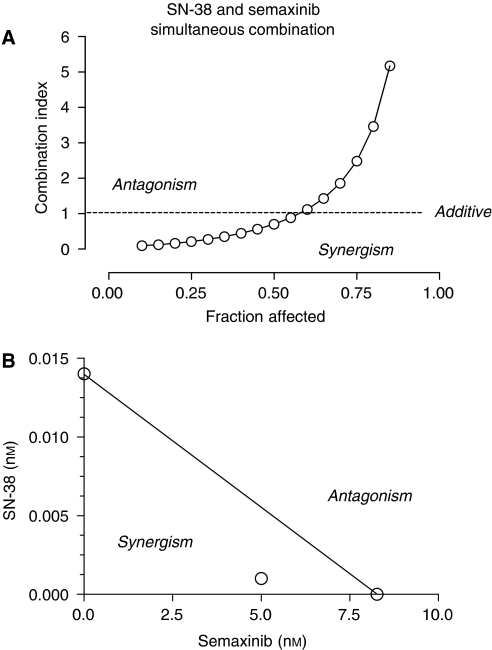
Combination index–fraction affected plot of semaxinib and SN-38 simultaneous 144-h combination in HMVEC-d cells (**A**). Isobologram analysis of HMVEC-d cell growth inhibition by simultaneous combination of semaxinib and SN-38 (**B**). The IC_50_ values of each drug are plotted on the axes; the solid line represents the additive effect, whereas the point representing the concentrations of semaxinib and SN-38 resulting in 50% growth inhibition of the combination is reported on the left of the connecting line, indicating synergism.

Simultaneous and continuous exposure of HMVEC-d cells to low concentrations of SN-38 and semaxinib for 144 h showed strong and moderate synergism (CI <1) at effect levels of 25 and 50% inhibition of cell proliferation, respectively, but an antagonistic effect for fraction affected higher than 65% (Figure 2A). Figure 2B shows a representative isobologram of HMVEC-d cells exposed to low concentrations of SN-38 and semaxinib for 144 h with simultaneous and continuous exposure schedule of treatment. The position of the data point on the left of the line connecting the IC_50_ values of SN-38 and semaxinib indicates synergism (Figure 2B).

#### Protracted low-dose treatment with SN-38 modulates expression and secretion of TSP-1 and VEGF in endothelial and cancer cells

**Figure 3:**
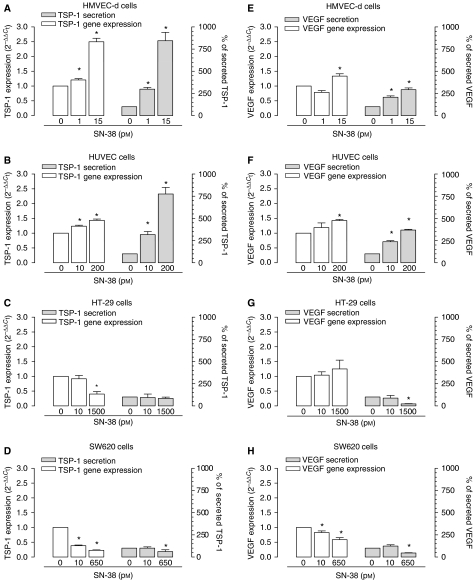
Thrombospondin-1 (TSP-1) gene expression and secretion in HMVEC-d (**A**), HUVEC (**B**), HT-29 (**C**) and SW620 (**D**) cells exposed to SN-38 at a concentration corresponding to the experimental IC_50_ of cell proliferation and at a lower and inactive concentration or with vehicle alone for 144 h. Vascular endothelial growth factor (VEGF) expression and secretion in HMVEC-d (**E**), HUVEC (**F**), HT-29 (**G**) and SW620 (**H**) cells exposed to the above-mentioned SN-38 concentrations. Columns and bars, mean values ±s.e., respectively. ^*^P<0.05 *vs* vehicle-treated controls. Thrombospondin-1 and VEGF concentrations in conditioned media were measured with EIA and ELISA kits, respectively, and they were normalised to total protein concentration.

SN-38 significantly increased TSP-1 expression in both endothelial cell populations tested at their respective IC_50_s and lower doses (*P*<0.05; Figure 3A and B). In particular, a 144-h SN-38 exposure at its IC_50_ levels resulted in a significant increase in TSP-1 expression in HMVEC-d cells (2.49±0.12 *vs* 1.0 of control expression; Figure 3A). Similar results were observed for HUVEC cells, in which TSP-1 expression was increased by SN-38 up to 1.44±0.05 *vs* 1.0 of controls (Figure 3B). In contrast, TSP-1 expression in HT-29 and SW620 cancer cells was stable (Figure 3C) or decreased at low SN-38 concentrations (Figure 3D), whereas at IC_50_ SN-38 levels, it was significantly decreased when compared to controls (Figure 3C and D). Moreover, SN-38 exposure positively modulated TSP-1 protein secretion (normalised for total protein) in both the endothelial cell populations tested (Figure 3A and B), confirming the data obtained at the mRNA level. In particular, TSP-1 secretion was significantly increased up to 845±94 and 775±74% in HMVEC-d and HUVEC cells *vs* 100% of controls, respectively, at the IC_50_ SN-38 concentrations (Figure 3A and B). Interestingly, results obtained with cancer cell cultures revealed that at SN-38 concentrations corresponding to their IC_50_s and at lower exposures, secreted TSP-1 was unchanged (Figure 3C) in HT-29 cells or significantly reduced (Figure 3D) up to 61.5±20 *vs* 100% of controls in SW620 cells.

The results of VEGF gene expression and protein secretion analysis showed a differential response among endothelial cell lines and colorectal cancer cells after 144-h SN-38 treatments. SN-38, at its IC_50_ levels, significantly increased VEGF expression both in HMVEC-d cells (1.34±0.08 *vs* 1.0 of control expression; Figure 3E) and HUVECs (1.43±0.04 *vs* 1.0 of control expression; Figure 3F); only a minimal and not significant enhancement of VEGF expression was observed in HT-29 cells (Figure 3G) whereas there was a significant decrease in VEGF mRNA in SW620 cells (0.59±0.07 *vs* 1.0 of control expression; Figure 3H). Although very low secreted levels of VEGF were measured (normalised for total protein), significant increments were observed at a SN-38 concentration corresponding to IC_50_ levels in both HMVEC-d and HUVEC cell lines (293±20 and 369±8.5 *vs* 100% of control, respectively; Figure 3E and F). On the contrary, VEGF secretion significantly decreased in both HT-29 cells (Figure 3G) and SW620 cells (Figure 3H) at their respective IC_50_ concentrations.

**Figure 4:**
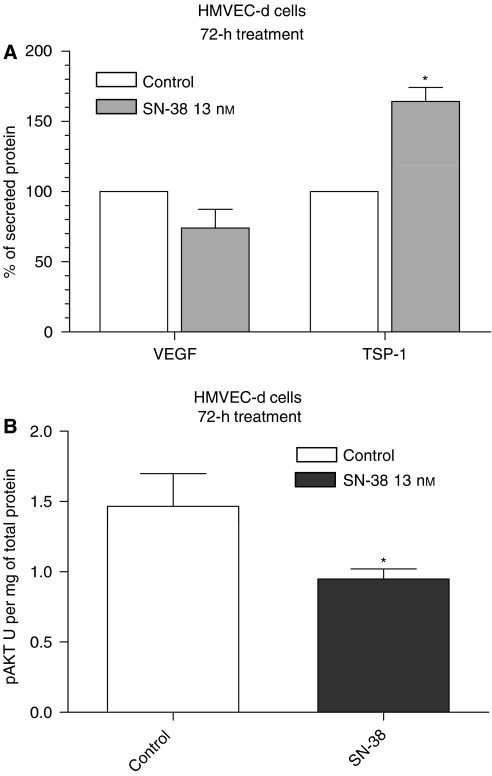
Thrombospondin-1 (TSP-1) and VEGF secretion in HMVEC-d (**A**) cells exposed to SN-38 at a concentration corresponding to the experimental IC_50_ of cell proliferation or with vehicle alone for 72 h. Thrombospondin-1 and VEGF concentrations in conditioned media were normalised to total protein concentration. Modulation of Akt (pThr^308^) phosphorylation by SN-38 in HMVEC-d cells after 72 h of treatment (**B**). Columns and bars, mean values ±s.e., respectively. ^*^P<0.05 *vs* vehicle-treated controls.

Interestingly, shorter periods of exposure to SN-38 (72 h) at concentrations that determined a 50% of inhibition of endothelial cell proliferation showed a significant increase of secreted TSP-1 (*P*<0.05) in conditioned media and a weak decrease in VEGF (*P*>0.05) (Figure 4A). Moreover, at the same SN-38 dose, the phosphorylation of Akt at Thr^308^ resulted in significant inhibition in HMVEC-d cells (Figure 4B).

### *In vivo* studies

#### Metronomic CPT-11 significantly inhibits HT-29 tumour growth inabsence of toxicity

**Figure 5:**
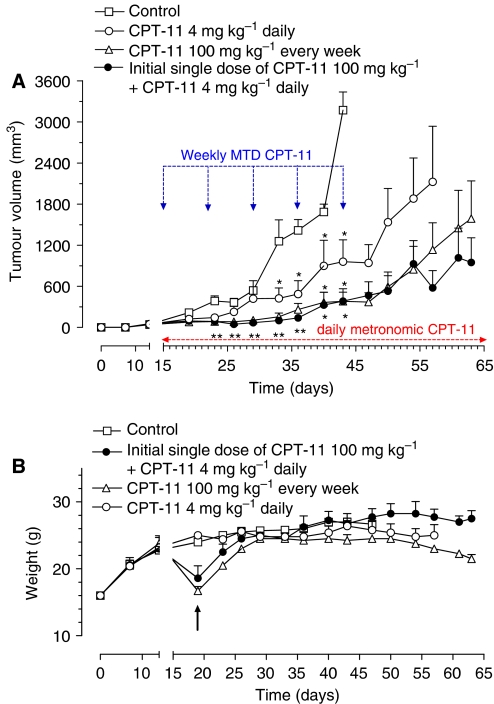
(**A**) Antitumour effect of (i) metronomic CPT-11 4 mg kg^−1^ i.p. daily, (ii) CPT-11 100 mg kg^−1^ i.p. every week (MTD schedule) and (iii) an initial single dose of CPT-11 100 mg kg^−1^ i.p. followed by metronomic CPT-11 4 mg kg^−1^ i.p. daily on HT-29 tumours xenotransplanted in CD *nu/nu* mice. ^*^P<0.05 with respect to controls. Symbols and bars, mean±s.e. (**B**) Body weight of HT-29 tumour-bearing control mice and mice treated with metronomic CPT-11, MTD CPT-11 and the single high dose followed by the metronomic schedule. MTD CPT-11 caused a severe loss of weight that required veterinary assistance with an immediate fluid therapy (↑; 0.9% saline, 40–80 ml kg^−1^ s.c. every 24 h) to save the animals. The group of animal at MTD CPT-11 needed a continued supportive fluid therapy for the duration of the treatment, whereas mice treated with a single initial dose of 100 mg kg^−1^ and metronomic maintenance did not need any additional fluid therapy until the end of the study. Symbols and bars, mean±s.e.

HT-29 cells injected s.c. in CD nu/nu mice grew quite rapidly, and tumour masses became detectable 10 days after xenotransplantation. Tumours in control animals showed a progressive enlargement in their dimensions and an exponential growth after ∼20 days; a mean volume of ∼3200 mm^3^ was reached at day 43 when the animals of the control group were killed (Figure 5A). Both MTD CPT-11 (100 mg kg^−1^ every week) and metronomic CPT-11 (4 mg kg^−1^ every day) were able to inhibit tumour growth, although to a different extent, and their therapeutic effect became significant starting on the eighth and the eighteenth day, respectively after the beginning of treatments as compared to controls (Figure 5A). The MTD schedule was effective, as expected, immediately, whereas the metronomic treatment effect was delayed; however, the toxicity profiles were very different between the two treatments (see below). It is noteworthy that in the group of animals receiving the initial single high CPT-11 dose followed by a maintenance therapy with metronomic CPT-11 until the end of the study, the reduction in tumour growth was significant by day 8 compared to controls (Figure 5A) and that the tumour growth curve showed a decrease during the 50-day schedule, significantly diverging from that of controls (e.g. at day 43, 383±181 *vs* 3172±263 mm^3^; *P*<0.05), comparable to CPT-11 MTD-treated animals, with a better response over the last 10 days of the experiment (Figure 5A).

Figure 5B shows the toxicity profiles of the three different schedules. The metronomic CPT-11 treatment was favourable and acceptable with no loss of weight throughout the course of the treatment (Figure 5B) whereas the MTD CPT-11 caused a severe loss of weight that necessitated veterinary assistance with an immediate fluid therapy (0.9% saline, 40–80 ml kg^−1^ s.c. every 24 h) to rescue the animals. The nadir of body weight loss was observed in the week following the first injection and the mean body weight of mice treated with doses of 100 mg kg^−1^ had returned to pretreatment values after 15 days with a continued supportive fluid therapy for the duration of the treatment (Figure 5B). However, mice treated with a single initial dose of 100 mg kg^−1^ and metronomic CPT-11 maintenance therapy recovered faster and did not require any additional fluid therapy until the end of the study (Figure 5B). Moreover, it is noteworthy that the MTD-treated animals never reached body weights similar to those treated with metronomic CPT-11 schedules.

#### Metronomic CPT-11 significantly decreases microvessel density and modulates VEGF and TSP-1 gene expression in HT-29 tumour tissues

**Figure 6:**
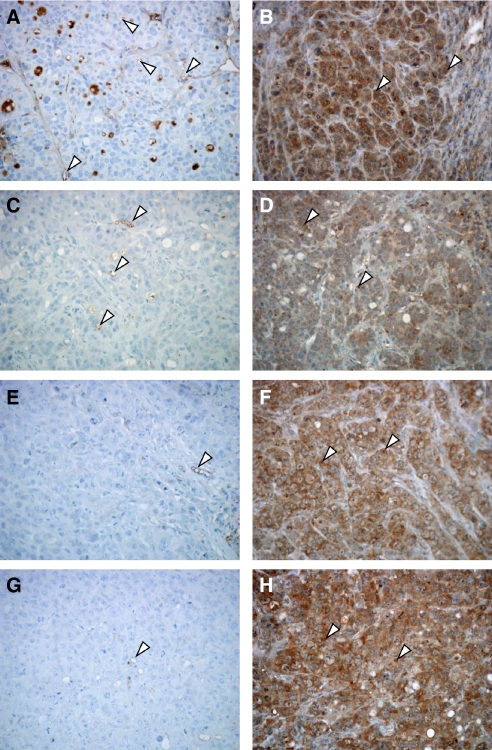
Representative images of immunohistochemistry of mouse CD31 and human VEGF in HT-29 xenografts in vehicle-treated mice (control group) (**A** and **B**, respectively), in CPT-11 100 mg kg^−1^ i.p. every week (MTD schedule) group of mice (**C** and **D**, respectively), in metronomic CPT-11 4 mg kg^−1^ i.p. daily group (**E** and **F**, respectively) and in the group treated with an initial single dose of CPT-11 100 mg kg^−1^ i.p. followed by metronomic CPT-11 4 mg kg^−1^ i.p. daily (**G** and **H**, respectively). Arrowheads, positively stained cells. Magnification, × 200.

The s.c. injection of HT-29 colorectal cancer cells produced a tumour, the histological picture of which, after staining with haematoxylin and eosin, was consistent with colorectal adenocarcinoma (data not shown). A well-defined CD-31 immunoreactivity was localised in endothelial cells inside the control tumour mass (Figure 6A). Representative microscopic pictures showed a reduction of microvessels in the MTD CPT-11-treated tumours (Figure 6C) whereas a clearly dramatic decrease of vessels in metronomic CPT-11-treated tumour samples was detected (Figure 6E and 6G). On the contrary, while control tumour xenografts showed a diffuse, strong and easily detectable immunoreactivity to the anti-VEGF antibody within cancer cells (Figure 6B), VEGF immunostaining weakly changed in MTD-treated samples (Figure 6D) and remained unaltered in the metronomic therapy-treated ones (Figure 6F and 6H).

**Figure 7:**
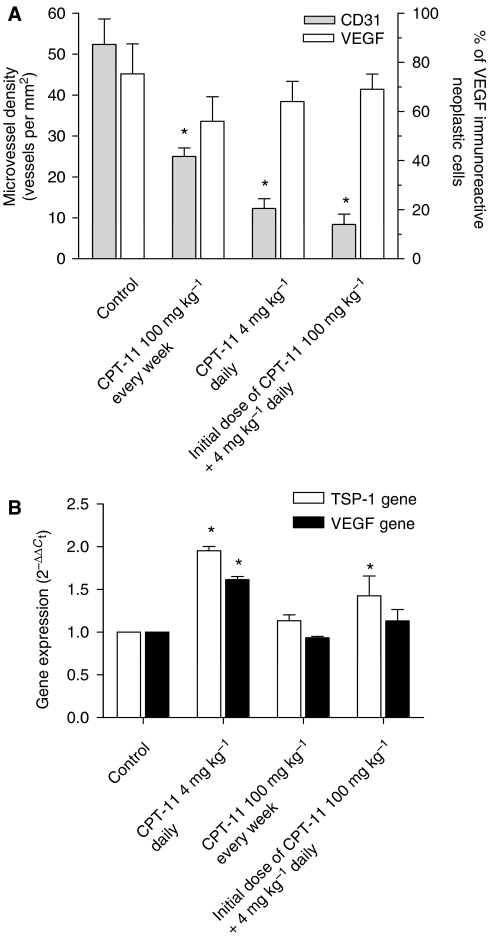
(**A**) Quantification of microvessel density and VEGF positivity in HT-29 tumour xenografts administered with MTD and metronomic CPT-11 schedules. (**B**) Thrombospondin-1 (TSP-1) and VEGF gene expression in explanted tumour samples from mice treated with MTD and metronomic CPT-11 schedules. Columns and bars, mean values ±s.e., respectively. ^*^P<0.05 *vs* vehicle-treated controls.

To quantify the observed differences among immunoreactivity of tissue samples of different animal groups, slides were analysed for vessel count in tumour sections stained with an antibody to CD31. Compared with the control treatment, MTD CPT-11 treatment resulted in a significant decrease in mean vessel count (25±2.1 *vs* 52.4±6.2 vessels per mm^2^ of controls; *P*<0.05; Figure 7A). Furthermore, both metronomic treatments (4 mg kg^−1^ daily and the one with the initial single 100 mg kg^−1^ dose followed by metronomic maintenance) resulted in a massive and significant reduction in mean vessel count (12.3±2.4 and 8.4±2.5 *vs* 52.4±6.2 vessels per mm^2^ of controls, respectively; *P*<0.05), although there was no statistical difference between them (Figure 7A). The quantification of cancer cell VEGF immunoreactivity confirmed that there was only a weak decrease in treated groups without reaching a significant difference (Figure 7A).

Figure 7B showed the modulation of human *TSP-1* and *VEGF* gene expression in treated tumours compared with vehicle-treated control tumours. A significant increase in both *TSP-1* and *VEGF* expression was found in the metronomic CPT-11-treated tumours (1.95±0.05 and 1.62±0.04 *vs* 1.0 of control expression; *P*<0.05) whereas no significant changes were demonstrated at the MTD dose (Figure 7B). Interestingly, these results suggested a discrepancy between *VEGF* gene expression and VEGF protein synthesis previously shown with immunohistochemistry and between TSP-1 expression *in vitro* and *in vivo* in the HT-29 cancer cell line.

#### Inhibition of tumour growth *in vivo* by metronomic irinotecan and semaxinib

**Figure 8:**
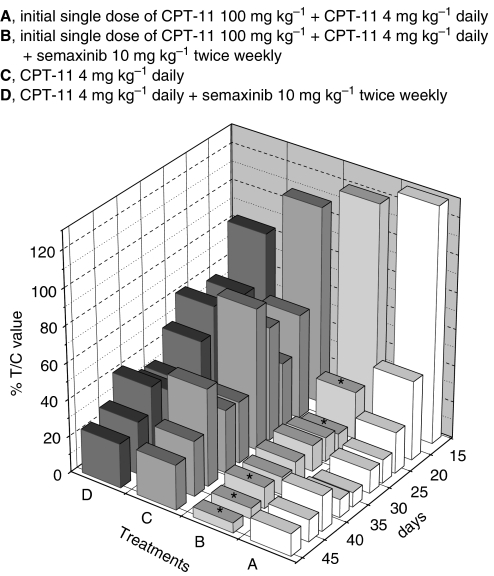
*In vivo* effects of the simultaneous combination of metronomic CPT-11 schedules and semaxinib on HT-29 tumours xenotransplanted in CD *nu/nu* mice expressed as % *T/C* value. Columns, % *T/C* values. ^*^P<0.05, initial single dose of CPT-11 100 mg kg^−1^ followed by metronomic CPT-11 4 mg kg^−1^ daily combined with semaxinib 10 mg kg^−1^ twice weekly *vs* initial single dose of CPT-11 100 mg kg^−1^ followed by metronomic CPT-11 4 mg kg^−1^ daily.

A significant *in vivo* antitumour effect of metronomic irinotecan was detected with both the adopted schedules (Figure 8); however, the administration of the initial single higher dose of irinotecan followed by the low-dose treatment confirmed to be more effective than the low-dose treatment alone (Figure 8). The tumour growth inhibition obtained with the combination of semaxinib 10 mg kg^−1^ twice weekly and metronomic irinotecan 4 mg kg^−1^ day^−1^ was almost superimposable with irinotecan alone and no statistical differences were noted (Figure 8). On the contrary, the combination of semaxinib 10 mg kg^−1^ twice weekly and the initial single dose of 100 mg kg^−1^ irinotecan followed by the 4 mg kg^−1^ day^−1^ irinotecan resulted in an almost complete regression of tumour volumes (*T/C* value 5.53% at day 47 of the experiment; Figure 8) and it was significantly different in the last 7 days of treatment from the same metronomic irinotecan schedule given alone (Figure 8). The toxicity profile was very favourable with no differences in loss of weight throughout the course of the experiment between single and combination treatments.

## DISCUSSION

Our translational study, for the first time, rationally demonstrated that low-dose metronomic irinotecan is effective in preclinical settings (*in vitro* and *in vivo*), as an antiangiogenic and antitumour schedule, modulating both *TSP-1* and *VEGF* gene expression and secretion.

The preferential antiangiogenic and pro-apoptotic activity *in vitro* for protracted low concentrations of chemotherapeutic drugs has been previously demonstrated for 4-hydroperoxycyclophosphamide, taxanes, epothilones and vinblastine (Bocci *et al*, 2002; Klement *et al*, 2002) but is unclear if all or other anticancer drugs have the same properties. Usually, *in vitro* experiments involved a single drug exposure of between 24 and 72 h; however, this does not mirror the *in vivo* situation when protracted low-dose metronomic chemotherapy protocols are administered daily. For this reason, we designed long-term *in vitro* assays in which human tumour cells and macrovascular or microvascular endothelial cells were exposed daily, for up to 6 days, to various low concentrations of SN-38. Our results are highly suggestive of the presence of the so-called ‘antiangiogenic window’ (Bocci *et al*, 2002) when protracted exposure to low concentrations of SN-38 was used. Indeed, there was a clear trend showing the effectiveness of SN-38 picomolar concentrations against endothelial cells, but not against tumour cells. This was the case not only for the inhibition of proliferation but also for the induction of apoptosis. These findings may be explained by the fact that *in vitro* SN-38 protracted treatment induced a significant increase of TSP-1 expression and secretion in the conditioned media of endothelial cells at effective concentrations (IC_50_). Moreover, TSP-1 has been previously described as a mediator of the specific antiangiogenic effects of this kind of therapy (Bocci *et al*, 2003; Damber *et al*, 2006), and also our results implicate this endogenous angiogenesis inhibitor as a major mediator of the low-concentration SN-38 antiendothelial *in vitro* effects we studied.

Interestingly, we also observed that SN-38 determined the significant increase of TSP-1 in conditioned medium of microvascular endothelial cells after 72 h of treatment, a change closer to the beginning of low-concentration therapy, and an inhibition of phosphorylation of Akt; these two observations may be mechanistically linked as demonstrated by Bussolati *et al* (2006), who described the modulatory role of Akt on TSP-1 synthesis in tumour endothelial cells (TEC). Indeed, the inhibition of Akt activation by administration of PI3K inhibitors significantly stimulated the synthesis and release of TSP-1 in TEC (Bussolati *et al*, 2006), in agreement with previous data by Niu *et al* (2004), who have shown that a loss of Akt signalling was related to a gradual increase in TSP-1 levels in endothelial cells.

Recently, it has been also reported that low doses of cyclophosphamide, cisplatin or docetaxel increase endothelial cell Fas receptor (Quesada *et al*, 2005; Yap *et al*, 2005). Thrombospondin-1 increases the level of Fas ligand on endothelial cells (Volpert *et al*, 2002; Yap *et al*, 2005). Moreover, it has been described that SN-38 induces Fas upregulation and caspase 8-mediated apoptosis in multiple myeloma cells (Catley *et al*, 2004). Thus, the low-dose SN-38 may also increase Fas ligand through the increase of TSP-1 secretion and, maybe, the Fas receptor such as other low doses chemotherapeutic drugs.

Moreover, these data provided our group with the prospect of exploiting TSP-1 expression or plasma levels as pharmacodynamic markers for CPT-11 metronomic chemotherapy clinical regimens, as previously suggested by Kerbel and Kamen (2004). Low concentrations of SN-38 did not significantly change VEGF expression in cancer cells; instead, high and antiproliferative concentrations of SN-38 caused a strong inhibition of VEGF in colorectal cancer cell lines, suggesting that SN-38-mediated cytotoxic damage of cancer cells can cause a strong inhibition of VEGF secretion. Interestingly, these results are similar to those obtained by Kamiyama *et al* (2005) in glioma cell lines treated with high SN-38 concentrations for 24 and 48 h (0.1 and 1 *μ*M).

To proceed with the development of metronomic CPT-11, the second step was to establish the antitumour and antiangiogenic activity of this schedule *in vivo*. On the basis of our *in vitro* IC_50_ data (a decrease of ∼70% from the lowest tumour cell IC_50_ to the highest endothelial IC_50_) and on data from previously published studies (Allegrini *et al*, 2004), we decided to treat animals with an MTD regimen (100 mg kg^−1^ weekly), a metronomic schedule (4 mg kg^−1^ day^−1^, i.e., a decreased total weekly dose of ∼70% *vs* the MTD schedule) and with an initial single high dose immediately followed by the metronomic regimen. The association of a bolus dose with a metronomic CPT-11 regimen was suggested by the results of Shaked *et al* (2005), who showed a superior antitumour activity combining intermittent high bolus doses (spaced 3–6 weeks apart) plus continuous low doses of cyclophosphamide in several tumour models, and by the work of Pietras and Hanahan (2005), who used a ‘chemo-switch’ regimen consisting of an initial short course of MTD cyclophosphamide, followed by long-term low-dose oral cyclophosphamide. Metronomic CPT-11 schedule was very well-tolerated, as expected from the experience with other low-dose chemotherapeutic drugs (Emmenegger *et al*, 2004), when compared to the MTD regimen, which required a continued supportive therapy, and significantly inhibited both tumour growth and neovascularisation. Moreover, the results suggested a superior efficacy and potent antitumour activity of the combined regimen; this enhanced activity was probably due to a direct effect of the irinotecan bolus dose on both drug-sensitive tumour cells and proliferating endothelial cells (Kerbel *et al*, 2000; Kamiyama *et al*, 2005) and a prolonged antiangiogenic effect of the metronomic regimen. Indeed, the microvessel density in tumour xenografts was markedly decreased in metronomic schedules when compared to control group and also to the MTD regimen, although to a lesser extent, confirming the data recently published by Ji *et al* (2007), who demonstrated an antiangiogenic effect of standard doses of CPT-11 in an orthotopic metastatic human colon cancer model in ND-GFP nude mice. A possible key role for TSP-1 in mediating some of the metronomic antiangiogenic effects of CPT-11 *in vivo* was suggested by the enhancement of the gene expression of the endogenous inhibitor in tumour xenografts found only in the low-dose-treated mice, whereas the VEGF immunohistochemistry analysis revealed no significant changes.

Further preclinical steps might be explored such as the combination of a targeted antiangiogenic drug, for example VEGFR-2 tyrosine kinase inhibitors, with metronomic irinotecan therapy, as such combinations show much greater antitumour efficacy compared with metronomic chemotherapy alone or the antiangiogenic drug alone (Klement *et al*, 2000; Kerbel and Kamen, 2004; Pietras and Hanahan, 2005). Indeed, to explore this possibility, we have associated SN-38 and irinotecan, *in vitro* and *in vivo* respectively, with semaxinib, a VEGFR-2 tyrosine kinase inhibitor. This combination obtained a synergistic effect *in vitro* and enhanced the antitumour activity *in vivo* of the association of a bolus dose with a metronomic irinotecan regimen. No preclinical data are currently available on irinotecan and semaxinib combination at standard doses; however, a phase I study of escalating doses of semaxinib and irinotecan in patients with advanced colorectal cancer has been recently published, describing signs of clinical activity without significant toxicity (Hoff *et al*, 2006). Our data confirmed the preclinical advantage of the association between an antiangiogenic drug and metronomic chemotherapy, as also recently shown by Ma and Waxman (2008), and may improve the possibility to translate a combination therapy based on metronomic irinotecan and VEGFR tyrosine kinase inhibitors into the clinics to reduce the toxicity and enhance the antitumour effects. Indeed, Garcia *et al* (2008) have recently reported encouraging phase II trial results of metronomic cyclophosphamide, administered daily, in combination with bevacizumab given every 2 weeks, for treatment of recurrent ovarian cancer.

In conclusion, the clinical development of a promising irinotecan-based metronomic chemotherapy and of related pharmacodynamic markers appears possible. The actual meaning of our results is the development of a rational and less- or nonempirical strategy for metronomic irinotecan chemotherapy ‘from bench to bed side’. Indeed, our study focused on and preclinically suggested possible approaches with respect to addressing the major questions regarding the clinical application of metronomic chemotherapy that relates to the actual antiangiogenic activity of the chosen chemotherapeutic drug, the dosing levels, the frequency of administration, the possible combinations with other drugs and the pharmacodynamic surrogate markers to be used. Indeed, based on our experimental data, we could suggest initiating pilot phase II clinical trials with at least these four characteristics: (i) an initial CPT-11 high dose followed by a long-term continuous infusion of irinotecan in colorectal cancer patients (including patients whose tumours are already resistant to irinotecan, but who may respond again because of the antiangiogenic effect of the metronomic schedules); (ii) the CPT-11 dose level should be reduced at least 70% of the maximum-tolerated total dose; (iii) the CPT-11 should be combined with a recommended dose of a VEGFR-2 tyrosine kinase inhibitor to improve the overall antitumour effects; (iv) the antiangiogenic activity could be assessed with pharmacodynamic parameters such as gene expression and plasma levels of TSP-1 and VEGF.
